# Ubiquinol-10 delays postovulatory oocyte aging by improving mitochondrial renewal in pigs

**DOI:** 10.18632/aging.102681

**Published:** 2020-01-20

**Authors:** Ying-Jie Niu, Wenjun Zhou, Zheng-Wen Nie, Dongjie Zhou, Yong-Nan Xu, Sun A. Ock, Chang-Guo Yan, Xiang-Shun Cui

**Affiliations:** 1 Department of Animal Science, Chungbuk National University, Cheongju, South Korea; 2 College of Agriculture, Yanbian University, Yanji, China; 3 Animal Biotechnology Division, National Institute of Animal Science, Rural Development Administration, Jeonju, South Korea

**Keywords:** ubiquinol-10, postovulatory aging, oxidative stress, mitochondria, pig

## Abstract

Ubiquinol-10, the reduced form of coenzyme Q10, protects mammalian cells from oxidative damage and enhances mitochondrial activity. However, the protective effect of ubiquinol-10 on mammalian oocytes is not well understood. In this study, we investigated the effect of ubiquinol-10 on porcine oocytes during postovulatory aging. Metaphase II oocytes were selected as fresh oocytes and further cultured for 48 h with different concentrations of ubiquinol-10 (0–400 μM) *in vitro* as a postovulatory aging model. After choosing the optimal concentration of ubiquinol-10 (100 μM) that maintained oocyte morphology and developmental competence during the progression of aging, the oocytes were randomly divided into five groups: fresh, control-24 h, ubiquinol-24 h, control-48 h, and ubiquinol-48 h. The results revealed that ubiquinol-10 significantly prevented aging-induced oxidative stress, GSH reduction, cytoskeleton impairment, apoptosis, and autophagy. Mitochondrial biogenesis (SIRT1 and PGC-1α) and mitophagy (PINK1 and PARKIN)-related proteins were decreased during aging. Addition of ubiquinol-10 prevented the aging-induced reduction of these proteins. Consequently, although mitochondrial content was decreased, the number of active mitochondria and ATP level were significantly increased upon treatment with ubiquinol-10. Thus, ubiquinol-10 has beneficial effects on porcine postovulatory aging oocytes owing to its antioxidant properties and ability to promote mitochondrial renewal.

## INTRODUCTION

Ubiquinol-10, the reduced form of coenzyme Q10, is a key electron transport mediator in the mitochondrial electron transport chain (ETC). It is located in the inner membrane of the mitochondria and transports electrons from complexes I and II to complex III [1]. Additionally, its functions in the cell membrane include antioxidant protection and it plays a role in redox homeostasis maintenance. Deficiency of ubiquinol-10 has been linked to the aging process [2]. It has been reported that ubiquinol-10 effectively protects lipid membranes and lipoproteins from oxidative damage in human polymorphonuclear leukocytes [3]. Moreover, it suppresses aging-related nuclear DNA and mitochondrial DNA (mtDNA) damage as well as mitochondrial deletion [4, 5] and defends against the age-induced decline in mitochondrial activity and function by upregulating SIRT1/PGC-1α axis-mediated mitochondrial biogenesis [6].

Following maturation, oocytes arrest at metaphase II (MII) and await fertilization in the oviduct of the female reproductive tract or in *in vitro* conditions. On occasions where fertilization does not occur within an optimal time frame, MII oocytes experience a process of degradation known as “postovulatory aging” [7]. Unfortunately, because no visual signs of ovulation are observed in humans and some mammals, if intercourse and ovulation are not synchronous, the likelihood of aged oocytes fertilizing with fresh spermatozoa *in vivo* may increase. However, in *in vitro* conditions, when assisted reproduction techniques are performed, prolonging the culture time is often unavoidable, which causes oocyte aging *in vitro*. Postovulatory aging induces many abnormal effects on cell biology, including partial exocytosis of cortical granules [8, 9], hardening of the zona pellucida [9, 10], decline in maturation-promoting factor (MPF) and MAPK levels [11], abnormalities in the cytoskeleton, and condensation of the chromosome [12]. It can also induce mitochondrial dysfunction leading to apoptosis [13–15], perturbation of Ca^2+^ homeostasis, oxidative damage to lipids, protein, and DNA components of the cell [16] as well as epigenetic changes [17]. Finally, postovulatory aging also reduces the fertilization rate and embryo quality and increases the likelihood of abnormalities in the offspring. In these aberrations, oxidative stress may initiate a cascade of other events during postovulatory degeneration of the MII oocyte [7]. Previous studies indicate that addition of antioxidants, such as β-mercaptoethanol, L-cysteine, resveratrol, or melatonin, to the oocyte culture medium may delay the process of postovulatory oocyte aging [7, 18–23].

The DNA, protein, and lipids of the mitochondria are located close to the ETC, which is the source of reactive oxygen species (ROS) [24, 25]. Hence, they are particularly susceptible to oxidative attack. Furthermore, the absence of protective histones and DNA repair mechanisms accelerates mtDNA damage and deletions. Unfortunately, damaged mitochondria may increase ROS production, leading to a vicious cycle, because oxidative stress reciprocally aggravates mitochondrial damage.

Postovulatory aging induces mitochondrial dysfunction or depletion in a variety of animals, including humans [26], pigs [27], and mice [15, 26, 28]. Thus, finding a new chemical that possesses both antioxidant and mitochondrial protection properties may effectively prevent the process of ovulatory oocyte aging. It has been reported that, owing to its antioxidative properties, the oxidized form of coenzyme Q10, ubiquinone-10, can protect against both maternal-induced oocyte aging and postovulatory oocyte aging in mice [21, 29]. However, ubiquinol-10 not only decreases oxidative stress but also enhances mitochondrial activity and function by upregulating mitochondrial biogenesis [6]. Moreover, ubiquinol-10 has better absorption, bioavailability, and tissue uptake than ubiquinone-10 [30–32]. Research related to ubiquinol-10 in the oocyte aging process has not yet been reported. Compared with those of other species, porcine oocytes are more similar to human oocytes in many respects, including the closed oocyte volume (120–125 μm in diameter), average time for oocyte maturation (40–44 h for pig, 40 h for human) [33], with similar core transcriptional network required to maintain pluripotency [34], and similar developmental stage of embryonic genome activation [33, 35]. Moreover, they both contain a large quantity of endogenous lipids [36, 37]. Thus, the porcine oocyte is a good model for human reproduction research and clinical assisted reproductive technology applications. Thus, we hypothesized that ubiquinol-10, a lipid soluble antioxidant, may affect the mitochondrial activity of oocytes and delay oocyte aging after ovulation in pigs.

Therefore, in this study, we investigated whether ubiquinol-10 could delay postovulatory oocyte aging in pigs and examined the associated underlying mechanism. During the postovulatory aging process, oocytes were treated with or without ubiquinol-10. Addition of 100 μM ubiquinol-10 could maintain oocyte morphology and developmental competence. It also prevented aging-induced mitochondrial dysfunction, cytoskeleton impairment, apoptosis, and autophagy by its antioxidant properties and ability to promote mitochondrial renewal.

## RESULTS

### Ubiquinol-10 rescued aging-induced fragmentation of porcine oocytes

**Figure 1:**
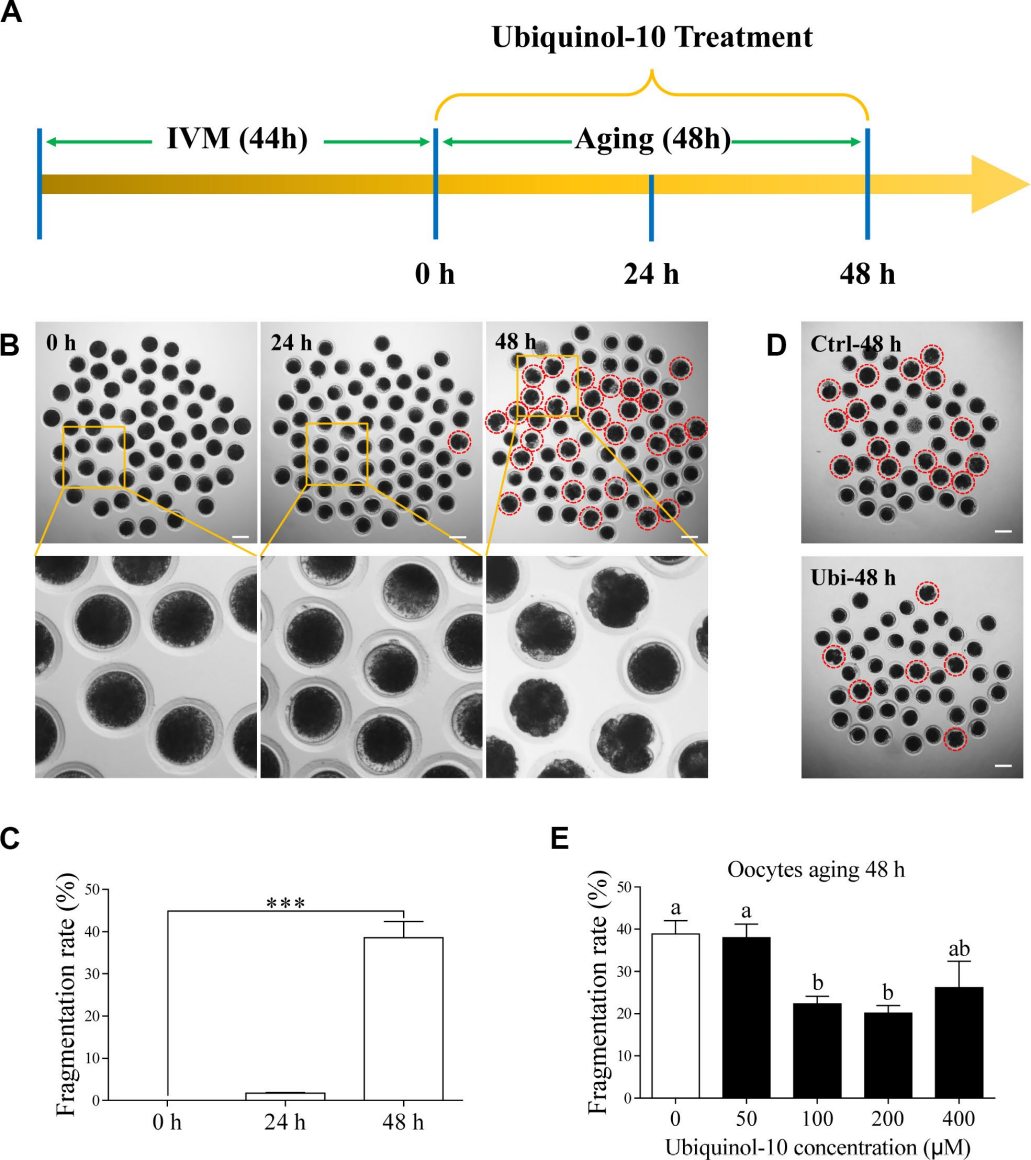
**Ubiquinol-10 rescued aging-induced fragmentation of porcine oocytes.** (**A**) Timeline diagram of postovulatory oocytes aging. Oocyte morphologies (**B**) and fragmentation rate (**C**) in aging 0 h, 24 h, and 48 h groups. The red dotted circle indicates fragmented oocytes. Original magnification, 3.5×. Scale bars represent 100 μm. ***p < 0.001 indicate significant differences. (**D**) Oocyte morphologies in Ctrl-48 h and 100 μM Ubi-48 h groups. The red dotted circle indicates fragmented oocytes. Scale bars indicate 100 μm. (**E**) The fragmentation rate of aging 48 h oocytes treated with different concentrations of ubiquinol-10 (0, 50, 100, 200, or 400 μM). Different letters indicate significant differences, p < 0.05. Ctrl-48 h: oocytes aging 48 h without ubiquinol-10. Ubi-48 h: oocytes aging 48 h with ubiquinol-10.

After *in vitro* maturation for 44 h, the MII oocytes were selected as fresh oocytes. Then, these oocytes were further cultured *in vitro* for 48 h as a postovulatory aging model (Figure 1A). The porcine oocyte morphology was observed at 0, 24, and 48 h of aging (Figure 1B). Here, 1.73 ± 0.11% oocytes were fragmented at 24 h of aging, but 38.54 ± 3.47 % oocytes were fragmented at 48 h of aging (p < 0.001, Figure 1C). To investigate whether ubiquinol-10 can maintain oocyte morphology during postovulatory aging, the oocytes were treated with ubiquinol-10 at five different concentrations (0, 50, 100, 200, and 400 μM) during postovulatory aging. The results showed that ubiquinol-10 treatment at 50 and 400 μM did not affect the fragmentation rate of the aging oocytes at 48 h (p > 0.05, 37.98 ± 2.65 % and 26.15 ± 5.14%); however, 100 and 200 μM ubiquinol-10 significantly reduced the fragmentation rate of the aging oocytes (p < 0.05, 22.37 ± 1.59 % and 20.14 ± 1.65%), compared with those in the control group (38.84 ± 2.91 %), at 48 h (p < 0.01, Figure 1D and 1E). Therefore, 100 μM ubiquinol-10 was selected for further studies.

### Ubiquinol-10 rescued aging-induced oxidative stress

**Figure 2:**
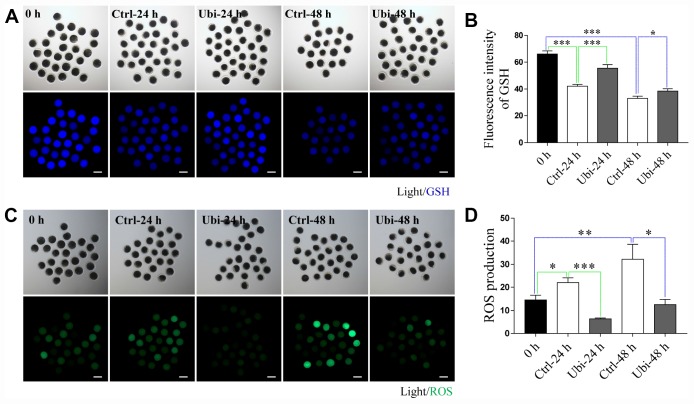
**Ubiquinol-10 rescued aging-induced oxidative stress.** Images (**A**) and GSH level (**B**) in 0 h, Ctrl-24 h, Ubi-24 h, Ctrl-48 h, and Ubi-48 h oocytes. Scale bars indicate 100 μm. Images (**C**) and ROS level (**D**) in 0 h, Ctrl-24 h, Ubi-24 h, Ctrl-48 h, and Ubi-48 h oocytes. Scale bars indicate 100 μm. *p < 0.05, **p < 0.01, and ***p < 0.001 indicate significant differences. 0 h: fresh oocytes. Ctrl-24 h: oocytes aging 24 h without ubiquinol-10. Ubi-24 h: oocytes aging 24 h with ubiquinol-10.

To determine the antioxidant protection effects of ubiquinol-10 on *in vitro* aging oocytes, the GSH and ROS levels were detected by the CMF_2_HC and H_2_DCF-DA reactions, respectively. As shown in Figure 2A and 2B, oocyte GSH levels gradually decreased during oocyte aging *in vitro* (p < 0.001). However, GSH levels in ubiquinol-10-treated oocytes were higher than those in oocytes not treated with ubiquinol-10 at both 24 and 48 h of aging (p < 0.05). ROS production substantially increased from 0 to 48 h in the aging oocytes *in vitro* (p < 0.05). However, ROS levels in the ubiquinol-10-treated oocytes were significantly lower than those in the control oocytes at both 24 and 48 h of aging (p < 0.05, Figure 2C and 2D). These data suggest that ubiquinol-10 can prevent GSH reduction and ROS production.

### Ubiquinol-10 rescued aging-induced mitochondrial dysfunction

**Figure 3:**
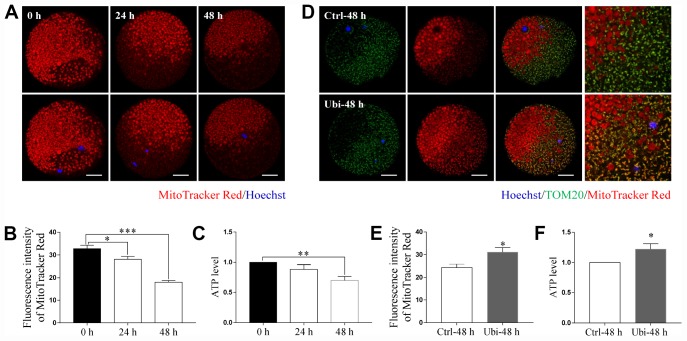
**Ubiquinol-10 rescued aging-induced mitochondrial dysfunction.** Images (**A**) and fluorescence intensity (**B**) of MitoTracker Red in aging 0 h, 24 h, and 48 h oocytes. Scale bars indicate 20 μm. (**C**) ATP levels of oocytes in aging 0 h, 24 h, and 48 h groups. (**D**) Colocalization of TOM20 and MitoTracker Red in Ctrl-48 h and Ubi-48 h oocytes. Scale bars indicate 20 μm. (**E**) Fluorescence intensity of MitoTracker Red in Ctrl-48 h and Ubi-48 h oocytes. (**F**) ATP levels in Ctrl-48 h and Ubi-48 h oocytes. *p < 0.05, **p < 0.01, and ***p < 0.001 indicate significant differences.

MitoTracker Red CMXRos was used to detect mitochondrial activity. As shown in Figure 3A and 3B, mitochondrial activity was significantly decreased during *in vitro* aging (p < 0.05). After 48 h of aging, the mitochondrial activity reduced by approximately 45% of the normal levels (p < 0.001). The ATP level was also measured as an estimate of mitochondrial function. As shown in Figure 3C, although the ATP level was not decreased after 24 h of *in vitro* aging (p > 0.05), it had considerably decreased after 48 h of aging *in vitro* (p < 0.01). Thus, to assess whether ubiquinol-10 could have beneficial effects on mitochondria during postovulatory aging of oocytes, oocytes were treated with ubiquinol-10 for 48 h during aging *in vitro* before measurement of mitochondrial activity and ATP levels. The results showed that the aging-induced reduction in mitochondrial activity and ATP level was noticeably prevented by the addition of ubiquinol-10 (p < 0.05, Figure 3D–3F). These findings indicate that ubiquinol-10 had protective effects on mitochondria of oocytes during postovulatory aging.

### Ubiquinol-10 rescued aging-induced reduction of mitochondrial biogenesis and mitophagy

**Figure 4:**
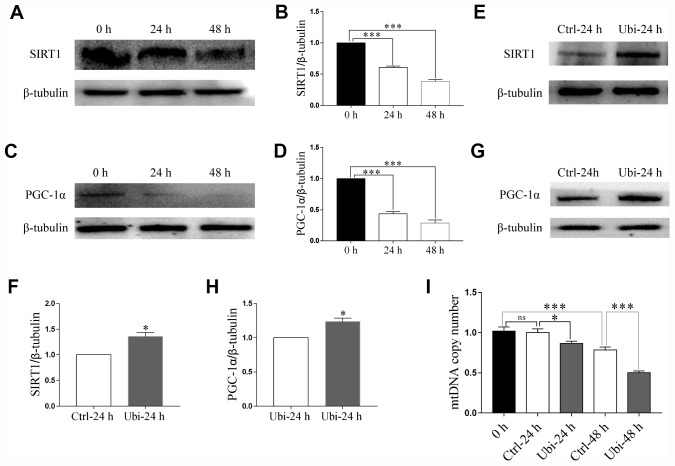
**Ubiquinol-10 rescued aging-induced reduction of mitochondrial biogenesis.** Protein levels of SIRT1 (**A** and **B**) and PGC-1α (**C** and **D**) in aging 0 h, 24 h, and 48 h oocytes. Protein levels of SIRT1 (**E** and **F**) and PGC-1α (**G** and **H**) in Ctrl-24 h and Ubi-24 h oocytes. (**I**) mtDNA copy number in 0 h, Ctrl-24 h, Ubi-24 h, Ctrl-48 h, and Ubi-48 h oocytes. ns, p > 0.05, *p < 0.05, and ***p < 0.001 indicate significant differences.

**Figure 5:**
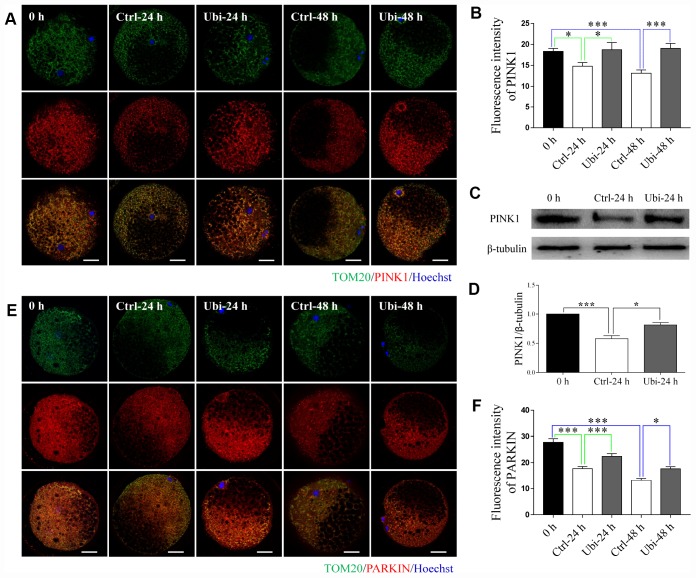
**Ubiquinol-10 rescued aging-induced compromise of mitophagy.** Immunofluorescence images (**A**) and relative fluorescence intensity (**B**) showing PINK1 expression in 0 h, Ctrl-24 h, Ubi-24 h, Ctrl-48 h, and Ubi-48 h oocytes. Scale bars indicate 20 μm. Protein levels of PINK1 (**C** and **D**) in 0 h, Ctrl-24 h, and Ubi-24 h oocytes were confirmed by western blotting. Immunofluorescence images (**E**) and relative fluorescence intensity (**F**) showing PARKIN expression in 0 h, Ctrl-24 h, Ubi-24 h, Ctrl-48 h, and Ubi-48 h oocytes. Scale bars indicate 20 μm.*p < 0.05 and ***p < 0.001 indicate significant differences.

To estimate whether mitochondrial biogenesis contributed to the beneficial effects of ubiquinol-10 on mitochondria in aging oocytes, two markers of mitochondrial biogenesis, SIRT1 and PGC-1α, were detected. Notably, the expression of both SIRT1 and PGC-1α was downregulated after 24 and 48 h of *in vitro* aging (p < 0.001, Figure 4A–4D). Treatment with ubiquinol-10 upregulated the expression of SIRT1 and PGC-1α compared with the control group without ubiquinol-10 treatment at 24 h of aging (p < 0.05, Figure 4E–4H). Next, we analyzed mtDNA copy number using RT-PCR. In agreement with previous results [38], the mtDNA copy number was considerably decreased at 48 h of aging (p < 0.001, Figure 4I). However, compared with the oocytes not treated with ubiquinol-10, the mtDNA copy number in ubiquinol-10-treated oocytes was significantly decreased at both 24 and 48 h of aging, rather than increased as we had predicted (p < 0.05, Figure 4I). Therefore, the expression of two mitophagy-related markers, PINK1 and PARKIN, was examined. As shown in Figure 5A–5F, the expression of both PINK1 and PARKIN was decreased during postovulatory aging (p < 0.05). Compared with the aging-only group, the expression of PINK1 and PARKIN was upregulated in the ubiquinol-10-supplemented groups both at 24 and 48 h of aging (p < 0.05). Collectively, our results show that ubiquinol-10 improved both mitochondrial biogenesis and mitophagy.

### Ubiquinol-10 rescued aging-induced cytoskeleton impairment

**Figure 6:**
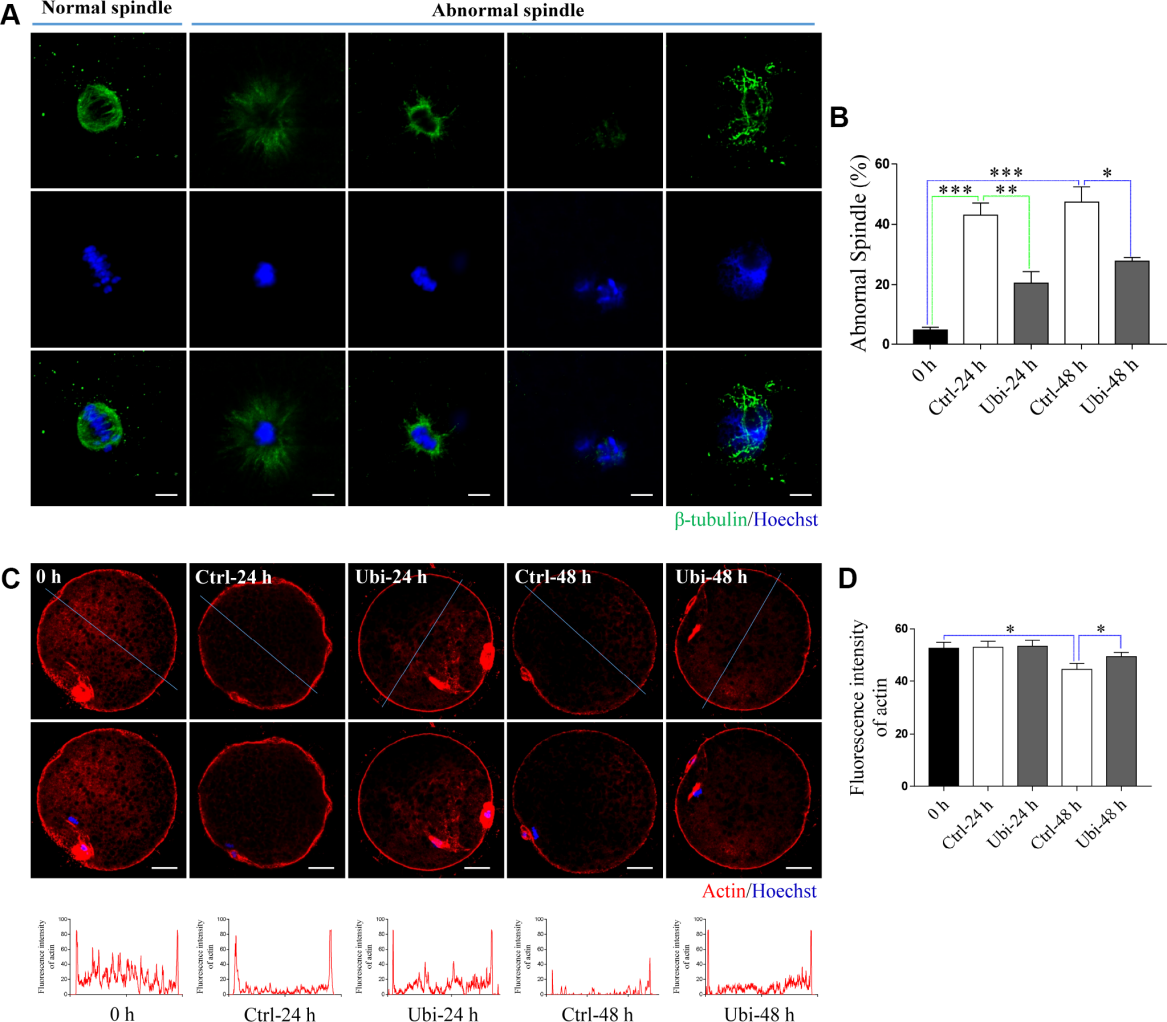
**Ubiquinol-10 rescued aging-induced cytoskeleton impairment.** (**A**) Representative confocal images of normal and abnormal spindles. Scale bars indicate 4 μm. (**B**) The ratio of abnormal spindle in 0 h, Ctrl-24 h, Ubi-24 h, Ctrl-48 h, and Ubi-48 h groups. Representative confocal images (**C**) and fluorescence intensity (**D**) of actin in 0 h, Ctrl-24h, Ubi-24 h, Ctrl-48 h, and Ubi-48 h groups. Scale bars indicate 20 μm. *p < 0.05 indicates significant differences.

Spindle elongation, dispersal, or disruption was revealed in previous studies [12, 39]. In our study, spindle integrity was observed by immunostaining. As shown in Figure 6A and 6B, aging caused an increase in abnormal spindles (p < 0.001); however, ubiquinol-10 decreased the rate of abnormal spindles, compared to the aging group, at both 24 and 48 h (p < 0.05). Actin was stained with phalloidin-TRITC. The actin signal was decreased in oocytes after 48 h of aging (Figure 6C). However, compared to the aging-only group at 48 h, ubiquinol-10 was able to maintain the expression of actin (Figure 6C). Statistical analysis showed that, compared to fresh oocytes, the intensity of actin was decreased after 48 h (P < 0.05) but not 24 h of aging (P > 0.05, Figure 6D). However, ubiquinol-10 can maintain the intensity of actin until 48 h of aging (Figure 6D).

### Ubiquinol-10 rescued aging-induced apoptosis and autophagy

**Figure 7:**
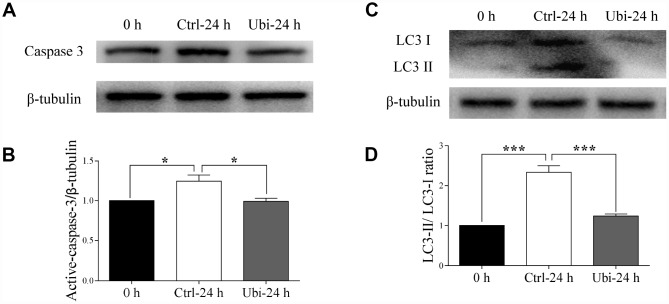
**Ubiquinol-10 rescued aging-induced apoptosis and autophagy.** Protein levels of active-caspase 3 (**A** and **B**) in 0 h, Ctrl-24 h, and Ubi-24 h oocytes. Protein levels of LC3 II (**C** and **D**) in 0 h, Ctrl-24 h, and Ubi-24 h oocytes. *p < 0.05 and ***p < 0.001 indicate significant differences.

As a result of the activation of caspases, the process of postovulatory aging culminates in apoptosis [15, 40]. Thus, to detect whether postovulatory aging was prevented, the expression of active-caspase 3 was examined by western blotting. As expected, the expression of active-caspase 3 was increased in the aging oocytes, compared to fresh oocytes (p < 0.05, Figure 7A and 7B). However, the addition of ubiquinol-10 prevented the aging-induced increase in active-caspase 3 expression at 24 h (p < 0.05, Figure 7A and 7B). In addition, postovulatory aging induced an increase in autophagy, which was prevented by supplementation with ubiquinol-10 at 24 h of aging (p < 0.001, Figure 7C and 7D). These results clearly demonstrate that ubiquinol-10 can rescue postovulatory aging-induced cell death and is a potent chemical that can delay postovulatory aging.

### Ubiquinol-10 rescued aging-induced effects on embryo development

**Figure 8:**
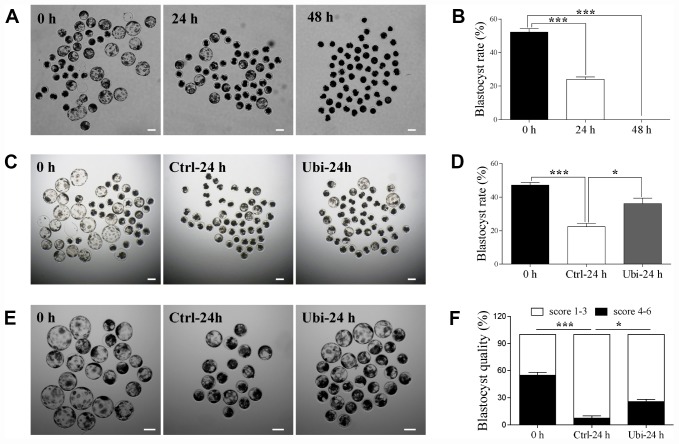
**Ubiquinol-10 rescued aging-induced compromise of embryo development.** The D6 embryo morphologies (**A**), blastocyst rate (**B**) in aging 0 h, 24 h, and 48 h groups. Scale bars indicate 100 μm. The D6 embryo morphologies (**C**) and blastocyst rate (**D**) in 0 h, Ctrl-24 h, and Ubi-24 h groups. Scale bars indicate 100 μm. (**E**) D6 blastocyst images in 0 h, Ctrl-24 h, and Ubi-24 h groups. (**F**) Blastocysts were graded on a scale from 1 to 6, as illustrated. Blastocyst quality was examined in 0 h, Ctrl-24 h, and Ubi-24 h groups. *p < 0.05 and ***p < 0.001 indicate significant differences.

Lastly, after parthenogenetic activation, the embryonic developmental competence was estimated by the blastocyst rate at day 6. The results showed that the blastocyst rate was considerably reduced after 24 h of aging (p < 0.001, 52.0 ± 1.9 % vs. 23.9 ± 1.2 %) and that no blastocyst was formed after 48 h of aging (p < 0.001, Figure 8A and 8B). Therefore, the protective capacity of ubiquinol-10 on the embryonic developmental competence of aging oocytes was detected after 24 h of aging. Compared with the aging group, the blastocyst rate was improved in the ubiquinol-10 treatment group (p < 0.05, 22.4 ± 0.4 % vs. 36.2 ± 2.8, Figure 8C and 8D). Moreover, the number of blastocysts of grade 4–6 were substantially decreased after 24 h of aging (p < 0.05, 54.8 ± 3.2 % vs. 7.5 ± 2.4 %); however, ubiquinol-10 supplementation (25.9 ± 2.4 %) significantly increased the blastocysts of grade 4–6 in comparison with the aging-only group (p < 0.05, Figure 8E and 8F). These results revealed that blastocyst formation and quality were both improved by the addition of ubiquinol-10.

## DISCUSSION

A widely accepted theory is that oxidative stress acts as a “trigger” for the process of postovulatory oocyte aging. Indeed, previous studies have indicated that antioxidant supplementation can delay the aging process in postovulatory oocytes [7, 18–23]. GSH, as an endogenous antioxidant, prevents the harmful effects of oxidative stress by reducing the accumulation of ROS in oocytes. However, ovulated MII oocytes exhibit a depletion of GSH and an accumulation of ROS in the condition that oocyte misses the optimal time frame for fertilization [27, 41]. An earlier study reported that dietary supplementation with ubiquinol-10 attenuates aging-induced oxidative damage to DNA, protein, and lipids and prevents the decline of GSH in senescence-accelerated mouse prone 1 (SAMP1) mice [6]. In our study, when the *in vitro* culture medium was supplemented with ubiquinol-10, we observed abated ROS production and depletion of GSH during the aging process of postovulatory oocytes. This data suggests that ubiquinol-10 can protect oocytes from oxidative stress during aging.

As a result of oxidative stress, a gradual decline in the concentration of MPF and MAPK within the postovulatory aging MII oocyte has been associated with an increased probability of spontaneous parthenogenetic activation and fragmentation [42]. Ubiquinol-10 not only prevented the oocyte fragmentation rate but also improved the developmental competence of the aging oocytes, including blastocyst formation and quality after activation. The cytoskeleton is another organelle affected by oxidative stress [27]. Abnormal spindle integrity and actin reduction are regularly associated with aging-induced oxidative damage. Ubiquinol-10 attenuated the abnormal spindle rate and prevented the downregulation of actin in the process of porcine oocyte postovulatory aging. In addition, oxidative stress induces autophagy and apoptosis in postovulatory aging oocytes. In our study, the addition of ubiquinol-10 prevented these processes. These findings demonstrate that ubiquinol-10 not only reduced the accumulation of ROS but also prevented the oxidative stress-induced deleterious effects on postovulatory aging oocytes. Combined with the fact that ubiquinol-10 increased the developmental competence of the aging oocytes, we believe that ubiquinol-10 can delay oocyte postovulatory aging in pigs.

Mitochondria are organelles particularly vulnerable to oxidative attack. In the present study, MitoTracker Red CMXRos was used to detect mitochondrial activity. This lipophilic cationic fluorescent dye can enter the mitochondria and accumulate inside this organelle owing to the negative mitochondrial membrane potential [43]. As previously reported, mitochondrial activity and ATP production were both reduced in the aging oocytes [44]; however, ubiquinol-10 supplementation was able to rescue these effects. The antioxidant properties of ubiquinol-10 may contribute to its beneficial effects on the mitochondria. Ubiquinol-10 also enhances mitochondrial biogenesis by upregulating SIRT1 and PGC-1α. This is another strategy by which ubiquinol-10 may play an important role in mitochondrial protection. Thus, we examined the expression of SIRT1 and PGC-1α and the mitochondrial amount. As expected, the expression of SIRT1 and PGC-1α was improved in the ubiquinol-10 treated aging oocytes, suggesting that ubiquinol-10 can enhance mitochondrial biogenesis during postovulatory oocyte aging. However, an interesting finding was that the mtDNA copy number was further decreased in the ubiquinol-10-supplemented group. Thus, we hypothesized that mitophagy, another important process that can affect mitochondrial amount, may be upregulated. To test this hypothesis, PINK1 and PARKIN, two important proteins associated with the process of mitophagy, were detected. In healthy mitochondria, PINK1 is translocated to the inner membrane of the mitochondria and degraded by the proteasome system [45]. However, in membrane potential-depolarized mitochondria, PINK1 accumulates in the outer membrane of the mitochondria [46] and then recruits PARKIN to the damaged mitochondrial outer membrane [47]. PARKIN is an E3 ubiquitin ligase; hence, the damaged mitochondrial proteins are then labeled by ubiquitin and directed for degradation by proteasomes or lysosomes [48]. Our results revealed that PINK1 and PARKIN were upregulated, indicating that mitophagy was also enhanced in the ubiquinol-10-supplemented group. Therefore, ubiquinol-10 can not only induce healthy mitochondrial production by promoting mitochondrial biogenesis but also accelerate the removal of damaged mitochondria by maintaining the process of mitophagy. Although the mitochondrial amount was slightly decreased in the ubiquinol-10-treated group, the remaining mitochondria were healthier. Thus, healthy mitochondria also contributed to the decreased ROS production. It is noteworthy that autophagy was downregulated when ubiquinol-10 sped up the mitophagy process. This may be because the primary reason for the increase in autophagy is the elimination of damaged cellular components, which were formed as a result of oxidative stress in the aging autophagy, as an adaptive response, was reduced. In this study, we reported that the mitochondrial quality control system was disrupted in postovulatory aging oocytes; supplementation with ubiquinol-10 can rescue these problems.

The protective effects of oxidized coenzyme Q10 have been well documented in mammalian oocytes [29, 49–52]. Supplementation with coenzyme Q10 could reduce oxidative stress and apoptosis and protect mitochondrial function and activity in *in vitro* conditions [29, 49–52]. These beneficial effects were also revealed in the present study: the reduced coenzyme Q10, ubiquinol-10, delayed porcine oocyte aging in *in vitro* conditions. However, the results suggested that mitochondrial biogenesis and mitophagy processes were also involved in the protection of mitochondria to promote mitochondrial renewal in porcine aging oocytes. Thus, further studies are needed to compare the difference between ubiquinone-10 and ubiquinol-10 in mammalian oocytes.

**Figure 9:**
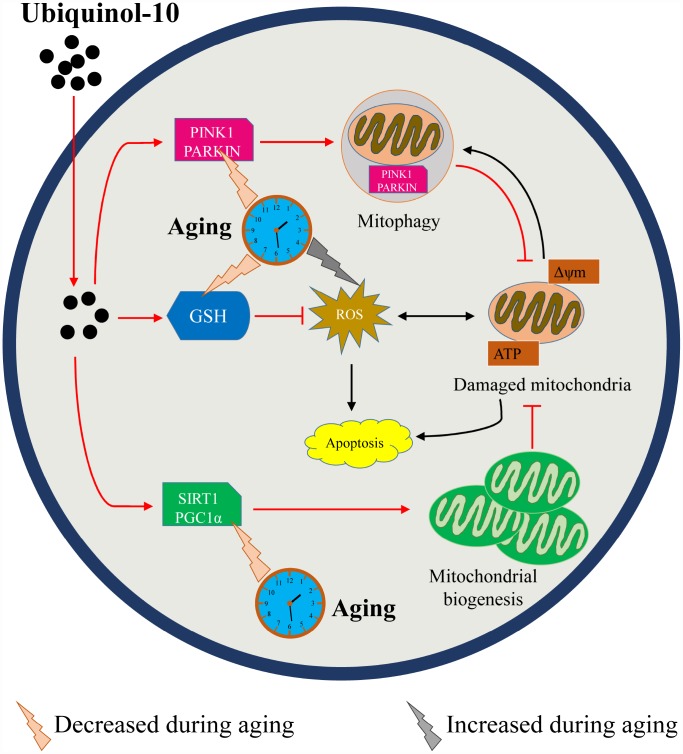
**Schematic representation of how ubiquinol-10 delays oocyte ovulatory aging by defending against oxidative stress and controlling mitochondrial quality.** Aging induced GSH depletion, ROS accumulation, mitochondrial biogenesis and mitophagy downregulation, followed by increases in autophagy and apoptosis. While supplementation with ubiquinol-10 prevented GSH depletion and ROS accumulation, it also enhanced mitochondrial biogenesis and mitophagy to control mitochondrial quality. Thus, ubiquinol-10 prevented postovulatory oocyte aging and promoted subsequent embryonic development in pigs. The black arrow indicates the aging-induced impairments in the oocytes. The red arrow indicates the beneficial effects of ubiquinol-10 on the aging oocytes.

As the post-ovulation time increases, GSH depletion and ROS accumulation may trigger a cascade of other events associated with oocyte aging, including oocyte morphology abnormalities, mitochondrial dysfunction, disturbed mitochondrial quality control system, increased autophagy and apoptosis, reduced cytoskeleton integrity, and compromised oocyte development. In aging oocytes, supplementing the media with ubiquinol-10 prevented GSH depletion and ROS accumulation, while enhancing mitochondrial biogenesis and mitophagy, to control mitochondrial quality (Figure 9). Thus, ubiquinol-10 prevented postovulatory oocyte aging and promoted subsequent embryonic development in pigs. These findings suggest that ubiquinol-10 could potentially be used to delay oocyte aging in other mammalian species or human oocytes processed for clinical assisted reproductive technology.

## MATERIALS AND METHODS

All chemicals were purchased from Sigma-Aldrich Corporation, Inc. (St. Louis, MO, USA), unless otherwise indicated. All manipulations were performed on a heated stage adjusted to 38.5°C, unless otherwise indicated.

### Collection of porcine oocytes and *in vitro* maturation

Ovaries from pre-pubertal gilts were collected from a local slaughterhouse (Farm Story Dodarm B&F, Umsung, Chungbuk, Korea) and transported to the laboratory at 37°C in saline supplemented with 75 mg/mL penicillin G and 50 mg/mL streptomycin sulfate. Follicles (3–6 mm in diameter) were aspirated using an 18-gauge needle connected to a 10-mL disposable syringe. Cumulus–oocyte complexes were selected according to their morphologic characteristics, i.e., those showing at least three layers of compact cumulus cells and evenly granulated ooplasm. After three rinses with maturation medium [TCM-199 (11150-059; Gibco, Grand Island, NY, USA) supplemented with 0.1 g/L sodium pyruvate, 10 ng/mL epidermal growth factor, 10% (v/v) porcine follicular fluid, 10 IU/mL luteinizing hormone, and 10 IU/mL follicle-stimulating hormone], approximately 50 cumulus–oocyte complexes were transferred into 4-well dishes containing 500 μL of maturation medium. The medium was covered with mineral oil, and the plates were incubated for 44 h at 38.5°C in a humidified atmosphere (5% CO_2_).

### *In vitro* aging and ubiquinol-10 treatment

After removing cumulus cells by repeated pipetting in 1 mg/mL hyaluronidase, only oocytes with first polar bodies were used for subsequent studies. For postovulatory oocyte aging, the collected MII oocytes were cultured in fresh in vitro maturation medium (IVM, mineral oil-covered) with or without ubiquinol-10 for an additional 24 or 48 h at 38.5°C in a humidified atmosphere (5% CO_2_). The fragmentation rate was checked at 24 and 48 h after aging *in vitro*.

### Parthenogenetic activation and *in vitro* culture

Fresh MII oocytes and aged oocytes were parthenogenetically activated using two direct-current pulses of 120 V for 60 μs in 297 mM mannitol (pH 7.2) containing 0.1 mM CaCl_2_, 0.05 mM MgSO_4_, 0.01% polyvinyl alcohol (PVA, w/v), and 0.5 mM HEPES. These oocytes were cultured in bicarbonate-buffered porcine zygote medium 5 (PZM-5) containing 4 mg/mL bovine serum albumin (BSA) and 7.5 μg/mL cytochalasin B for 3 h to suppress the extrusion of the pseudo-second polar body. Next, the oocytes were thoroughly washed and cultured in bicarbonate-buffered PZM-5 supplemented with 4 mg/mL BSA in 4-well plates for 6 days at 38.5°C (5% CO_2_). Blastocyst formation rate was examined at day 6. After 6 days of culture, the quality of the blastocysts was evaluated according to Gardner’s criteria [53]. Blastocysts were graded on a scale from 1 to 6 depending on their degree of expansion: Grade 1, blastocyst cavity occupies less than half of the blastocoel volume of the embryo; Grade 2, blastocyst cavity occupies more than half of the blastocoel volume of the embryo; Grade 3, blastocyst cavity completely fills the embryo; Grade 4, blastocyst cavity occupies more than the blastocoel volume of the embryo, with a thinning zona pellucida; Grade 5, hatching out of the zona pellucida; and Grade 6, hatched out of the zona pellucida.

### Glutathione (GSH) and ROS measurements

To measure GSH levels, oocytes were incubated at 37°C for 30 min in PBS/PVA containing 10 μM 4-chloromethyl-6,8-difluoro-7-hydroxycoumarin dye (CellTracker^TM^ Blue CMF_2_HC, Thermo Fisher Scientific, Waltham, USA) and then washed thrice with PBS/PVA. To measure ROS levels, oocytes were incubated at 37°C for 15 min in PBS/PVA containing 10 μM 2′,7′-dichlorodihydrofluorescein diacetate (H_2_DCF-DA, Cat # D399, Molecular Probes, Eugene, OR, USA) and then washed thrice with PBS/PVA. Fluorescence signals were captured as a TIFF file using a digital camera (DP72; Olympus, Tokyo, Japan) connected to a fluorescence microscope (IX70, Olympus). GSH and ROS levels were quantified by analyzing the fluorescence intensity in the oocytes using ImageJ software, version 1.44g (National Institutes of Health, Bethesda, MD, USA).

### Immunofluorescence and confocal microscopy

After washing thrice with PBS/PVA, embryos were fixed in 3.7% paraformaldehyde for 30 min at room temperature (20-25°C), permeabilized with PBS/PVA containing 0.5% Triton X-100 at room temperature for 30 min, and incubated in PBS/PVA containing 1.0% BSA at room temperature for 1 h. These embryos were incubated overnight at 4°C with anti-TOM20 (1:100, F-10, Cat # SC-17764, Santa Cruz Biotechnology), anti-PINK1 (1:100; Cat # 23707, Abcam), anti-β-Tubulin (1:100, D-10, SC-5274, Santa Cruz Biotechnology), or anti-PARKIN (1:100; Cat # 2132, Cell Signaling Technology) diluted in blocking solution. After washing thrice with PBS/PVA, the embryos were incubated at room temperature for 1 h with Alexa Fluor 488^TM^ Donkey anti-Mouse IgG (H+L) (1:200; Cat # A21202, Invitrogen) or Alexa Fluor 546^TM^ Donkey anti-Rabbit IgG (H+L) (1:200; Cat # A10040, Invitrogen). Actin was stained with phalloidin-TRITC at 4 °C for 4 h. The oocytes and embryos were stained with 10 μg/mL Hoechst 33342 for 10 min, washed thrice with PBS/PVA, mounted onto slides, and examined using a confocal microscope (Zeiss LSM 710 META). Images were processed using Zen software (version 8.0, Zeiss).

### Active mitochondrial staining

Oocytes were incubated at 38.5°C with 500 nM MitoTracker Red CMXRos (Cat #M7512, Invitrogen) for 30 min. After three washes with IVM, staining of TOM20 was carried out as described in the Immunofluorescence and confocal microscopy methods subsection.

### MtDNA copy number measurements

A pool of five oocytes was transferred to a 0.2-mL tube containing 8 μL lysis buffer (20 mM Tris, 0.4 mg/mL proteinase K, 0.9% Nonidet-40, and 0.9% Tween 20) and incubated at 65 °C for 30 min, followed by further incubation at 95 °C for 5 min. Samples were diluted 1:25 in sterile ddH_2_O before analysis. Subsequently, real-time PCR (RT-PCR) was performed as described in the Real-time RT-PCR methods subsection.

### ATP measurements

ATP content was measured using a luciferin–luciferase ATP assay system with a luminometer (CentroPRO LB 962; Berthold, ND, USA) according to the manufacturer instructions of the ATP determination kit (A22066, Molecular Probes). Briefly, 20 oocytes were collected into a 0.2-mL centrifuge tube containing 30 μL lysis buffer (20 mM Tris, 0.9% Nonidet-40, and 0.9% Tween 20). These embryos were homogenized by vortexing until lysis occurred. A standard reaction solution was prepared according to the manufacturer’s instructions and placed on ice in the dark before use. Before measurement, 5-μL samples were added to 96-well plates and equilibrated for 10 s. Subsequently, 200 μL of the standard reaction solution was added into each well, and the light signal was integrated for 10 s after a delay of 2 s. The light intensity in the control group was arbitrarily assigned a value of 1, and the light intensity in the treatment group was then measured and expressed as relative values for the control group.

### Western blot analysis

In total, 150 porcine oocytes per group were placed in 1x SDS sample buffer and heated at 98°C for 10 min. Proteins were separated by SDS-PAGE and transferred to polyvinylidene fluoride membranes. Next, the membranes were blocked in TBS containing 0.1% Tween 20 and 5% non-fat milk for 1 h and then incubated at 4°C overnight with anti-SIRT1 (1:1000; 13161-1-AP, Proteintech), PGC-1α (1:1000, 66369-1-Ig, Proteintech), PINK1 (1:1000; 23707, Abcam), LC3A/B (1:1000, 66139-I-IG, Proteintech), active-caspase 3 (1:1000, C8487, sigma), or β-Tubulin (1:1000, D-10, SC-5274, Santa Cruz Biotechnology). Then, the membranes were incubated at room temperature for 1 h with horseradish peroxidase-conjugated goat anti-mouse IgG or goat anti-rabbit IgG (1:1000; Santa Cruz Biotechnology). Blots were visualized using a CCD camera and UVI Soft software (UVITEC Cambridge).

### Statistical analysis

Each experiment was repeated at least three times, and representative images are shown in the figures. Data were examined using one-way analysis of variance (ANOVA) or Student’s *t*-test. All percentage data were subjected to arcsine transformation prior to statistical analysis and are presented as the mean ± SEM. Significance was set at p < 0.05. All calculations were performed using SPSS software v.19 (SPSS, Inc., Chicago, IL, USA).
